# Targeting Integrin β3: novel antiplatelet lignans 6’-Hydroxyjusticidin B and Neojusticin A from *Justicia procumbens* unveiled via multi-omics and biophysical validation

**DOI:** 10.1007/s13659-025-00577-w

**Published:** 2026-02-03

**Authors:** Meixian Xiang, Songtao Wu, Hanxiang Mei, Xiang Zheng, Cong Wang

**Affiliations:** 1 School of Pharmaceutical Sciences, South-Central Minzu University, Wuhan, 430074 China; 2 Hubei Key Laboratory of Resources and Chemistry of Chinese Medicine, School of Pharmacy, Hubei University of Chinese Medicine, Wuhan, 430065 China; 3 Hubei Shizhen Laboratory, Wuhan, 430061 China

**Keywords:** Justicia procumbens, Lignans, Antiplatelet aggregation, ITGB3, Molecular mechanism

## Abstract

Thrombotic disorders remain a global health burden, necessitating novel antiplatelet agents with improved safety and efficacy. This study investigates the molecular mechanisms of two lignans, 6'-Hydroxyjusticidin B (6'-HJB) and Neojusticin A (Neo-A), isolated from *Justicia procumbens* L., through an innovative target-driven strategy integrating LC/MS, proteomics, network pharmacology, and biophysical validation. For the first time, integrin β_3_ (ITGB3) was identified as their direct molecular target, with microscale thermophoresis (MST) confirming high-affinity binding, the dissociation constant (Kd) = 0.0642 ± 0.005 μM for 6'-HJB; 0.0097 ± 0.001 μM for Neo-A. This study not only elucidates the structural basis of their activity-C-6 hydroxylation in 6'-HJB enhances ITGB3 specificity, whereas Neo-A’s fused furan ring optimizes COX-1 interaction, but also establishes a paradigm shift from phenotypic screening to target-validated natural product research. The findings position 6'-HJB and Neo-A as promising candidates for the development of safer, ITGB3-mediated antithrombotic therapies, with future efforts directed toward structural optimization and preclinical validation.

**Graphical Abstract:**

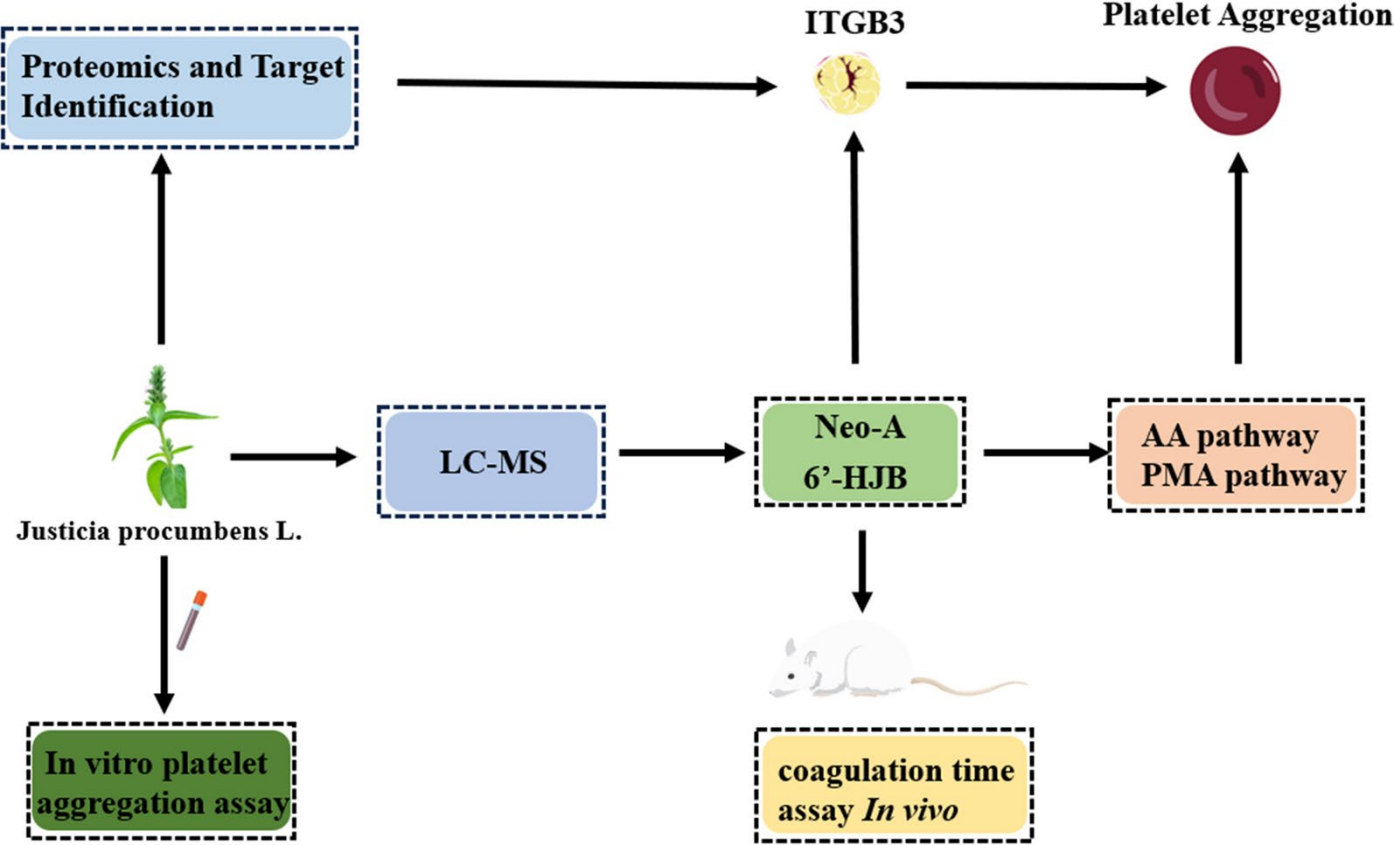

## Introduction

Thrombotic disorders, including myocardial infarction and ischemic stroke, remain the leading causes of mortality and morbidity worldwide, accounting for over 11 million deaths annually [1]. Platelet hyperactivity plays a central role in thrombogenesis [2], driven by complex signaling networks involving ITGB3, thromboxane A2 (TXA2), and adenosine diphosphate (ADP) receptors [3]. Current antiplatelet therapies, such as aspirin (cyclooxygenase inhibitor) and P2Y12 antagonists (clopidogrel, ticagrelor), reduce thrombotic risks but are limited by gastrointestinal toxicity, hemorrhage, and acquired resistance [4]. Approximately 15–30% of patients receiving aspirin exhibit incomplete platelet inhibition, necessitating the development of novel agents with distinct mechanisms [5]. While ITGB3 remains a pivotal target for suppressing platelet aggregation, limitations of current small-molecule inhibitors, like tirofiban-including transient efficacy, thrombocytopenia risk, and bleeding complications due to non-selective receptor blockade-drive the pursuit of advanced therapeutic modalities [6, 7]. Recent clinical innovations focus on engineering α_IIb_β_3_-targeted agents with improved specificity [8]. A bispecific antibody simultaneously engaging α_IIb_β_3_ and endothelial PECAM-1 has demonstrated thrombus-selective platelet inhibition in preclinical models, reducing bleeding propensity by 40% compared to conventional inhibitors [9]. This strategy spatially confines antithrombotic activity to activated platelets at vascular injury sites, circumventing systemic hemostatic disruption. Concurrently, pharmacogenomic insights reveal that ITGB3 genetic variants modulate interindividual responses to antiplatelet therapies, positioning this integrin as both a direct intervention target and a biomarker for personalized antithrombotic regimens [10]. Such advances underscore the untapped potential of α_IIb_β_3_ in balancing efficacy and safety [11].

Natural products derived from traditional Chinese medicine (TCM) offer a promising source of novel antithrombotic agents due to their multi-targeted action and reduced side effects [12]. *Justicia procumbens* L. (Acanthaceae), a commonly used TCM known as “Xia Ye Zhi Zi Cao”, has been traditionally employed to treat hematological disorders and inflammation [13]. Phytochemical investigations have identified lignans as the major bioactive components, including 6'-HJB and Neo-A. Previous studies have reported that its ethyl acetate extract exhibits dose-dependent antiplatelet activity in vitro and a half-maximal inhibitory concentration (IC₅₀) = 1.20 mg/mL and prolongs clotting time in mice, yet the bioactive components and underlying mechanisms remain elusive [14].

Lignans are a class of phenylpropanoid dimers with diverse pharmacological activities [15, 16], including antioxidant, anti-inflammatory, and anticancer effects [17]. Their structural diversity—characterized by 1- or 4-phenylnaphthalide skeletons—dictates target specificity and bioactivity [18, 19]. For instance, podophyllotoxin (a 4-phenylnaphthalide) inhibits topoisomerase II [20], while arctigenin (a dibenzylbutyrolactone lignan) modulates NF-κB signaling. 1—Phenylnaphtholide lignans may form specific interactions with platelet membrane proteins due to their unique hydroxyl substitution pattern. However, the antithrombotic potential of 1-phenylnaphthalide lignans remains unexplored [21].

ITGB3, a transmembrane glycoprotein critical for platelet aggregation and thrombus formation, has emerged as a validated target for antithrombotic drug development [22]. Upon platelet activation, ITGB3 undergoes conformational changes, enabling fibrinogen binding and platelet cross-linking [23]. Monoclonal antibodies targeting α_IIb_β_3_ (e.g., abciximab) effectively block thrombus formation but carry significant bleeding risks [24]. Small-molecule inhibitors, such as tirofiban, demonstrate improved safety profiles but require parenteral administration [25]. Identifying orally bioavailable small molecules with higher specificity and safety profiles is therefore critical.

## Result

### In vitro antiplatelet aggregation activity

**Fig. 1:**
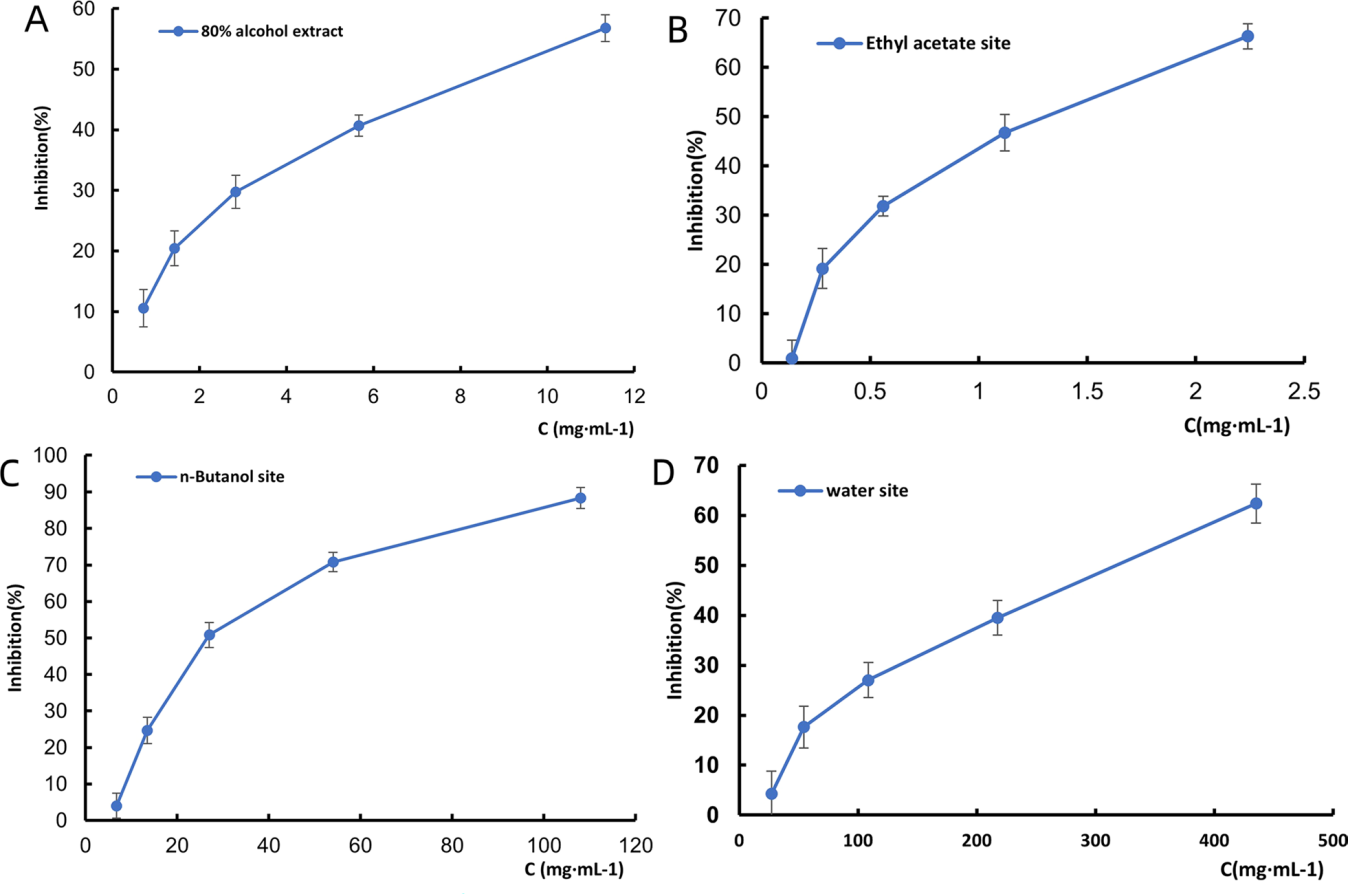
The ethyl acetate fraction showed the highest inhibitory potency against ADP-induced platelet aggregation. **A**–**D** Inhibition curves of 80% ethanol, ethyl acetate, n-butanol, and aqueous extracts on ADP-induced platelet aggregation (n = 3)

The ethyl acetate fraction demonstrated the highest inhibitory potency against ADP-induced platelet aggregation among all fractions tested from the *Justicia procumbens* L. (Fig. 1). The IC₅₀ was determined to be 1.20 ± 0.15 mg/mL, which was significantly lower than those of the n-butanol fraction (29.50 ± 2.83 mg/mL, P < 0.001) and aqueous fraction (284.19 ± 15.67 mg/mL, P < 0.001) (Fig. 1B). Kinetic analysis revealed that the inhibitory effect of the ethyl acetate fraction was time-dependent, with the maximum aggregation inhibition rate reaching 69.74% ± 2.29% after a 20-min incubation period (Fig. 1B–D). This time point was selected based on pre-experiments showing an optimal balance between drug-target interaction and platelet viability.

### Prolongation of clotting time in vivo

**Fig. 2:**
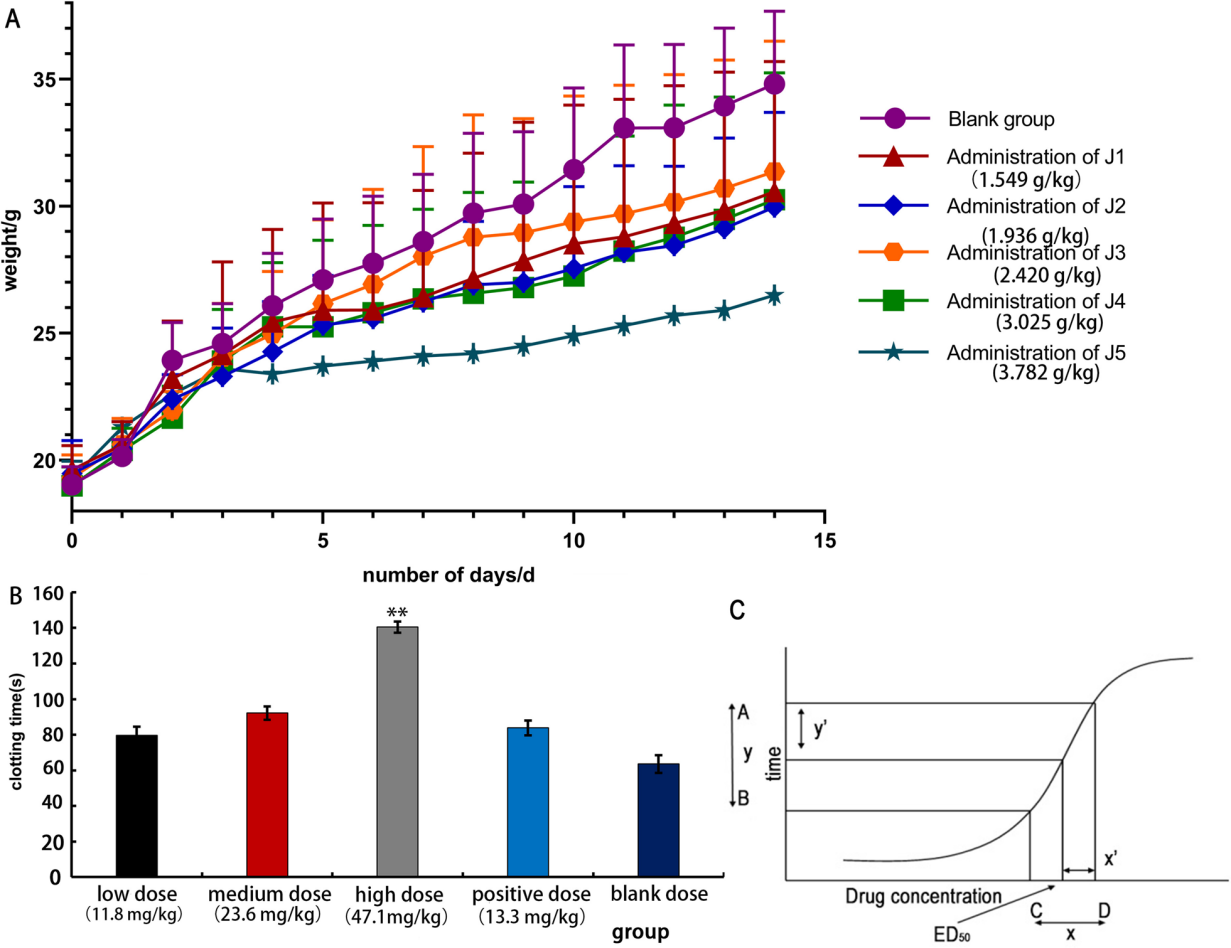
The ethyl acetate extract of *Justicia procumbens* L. is effective and has low toxicity. **A** Effect of body weight on acute toxicity test in mice. **B** Effect of blood clotting time in mice by ethyl acetate of *Justicia*, low dose (11.8 mg/kg), medium dose (23.6 mg/kg), high dose (47.1 mg/kg), aspirin group (13.3 mg/kg). Comparison with aspirin group: P < 0.01, *P < 0.05, **P < 0.01, ***P < 0.001. **C** The dose–response curve

After gavage administration, high-dose mice exhibited lethargy, weakness, and unsteady gait, followed by hyperactivity, body twisting, bradypnea, and tail cyanosis. Mortality occurred primarily within 2 h. Surviving mice fully recovered within 24 h. The low-dose group showed milder symptoms. Necropsy revealed no significant abnormalities in major organs except for renal pallor and whitish spotting. The data after gavage administration of the ethyl acetate extract of *Justicia procumbens* L. is shown in Fig. 2A. It can be seen that the body weight of the administered group increased more slowly than that of the blank group, and the trend of body weight growth of the surviving animals in all groups was similar to that of the blank group after 14 days of feeding. In the murine model, oral administration of the ethyl acetate extract at a high dose (47.1 mg/kg) resulted in a dose-dependent prolongation of clotting time (Fig. 2B). Specifically, the clotting time was extended to 140.4 ± 3.07 s (P < 0.01 vs. vehicle control), which was 1.8-fold longer than the control group (63.5 ± 5.00 s).

The results for the acute toxicity LD_50_ in mice are shown in Table 1. The therapeutic index (TI), calculated as the ratio of LD₅₀ to ED₅₀, was 84.3, indicating a favorable safety profile. In comparison, the positive control group treated with aspirin (13.3 mg/kg) showed a clotting time of 83.8 ± 4.1 s (P < 0.01 vs. control), confirming the validity of the assay. The ED₅₀ was determined according to the novel geometric method, which applies the principle of a right-angled triangle to the dose–response curve (Fig. 2C).

**Table 1:** Results of the acute toxicity test

Group	Dose (g/kg)	Herbal dosage (g/kg)	X	n	p	p2
J1	1.549	129.083	0.1901	10	0.1	0.01
J2	1.936	161.333	0.2869	10	0.4	0.16
J3	2.420	201.667	0.3838	10	0.5	0.25
J4	3.025	252.083	0.4807	10	0.6	0.36
J5	3.782	315.167	0.5777	10	0.9	0.81
			I = 0.097		Ʃp = 2.5	Ʃp^2^ = 1.59

### LC–MS analysis

**Fig. 3:**
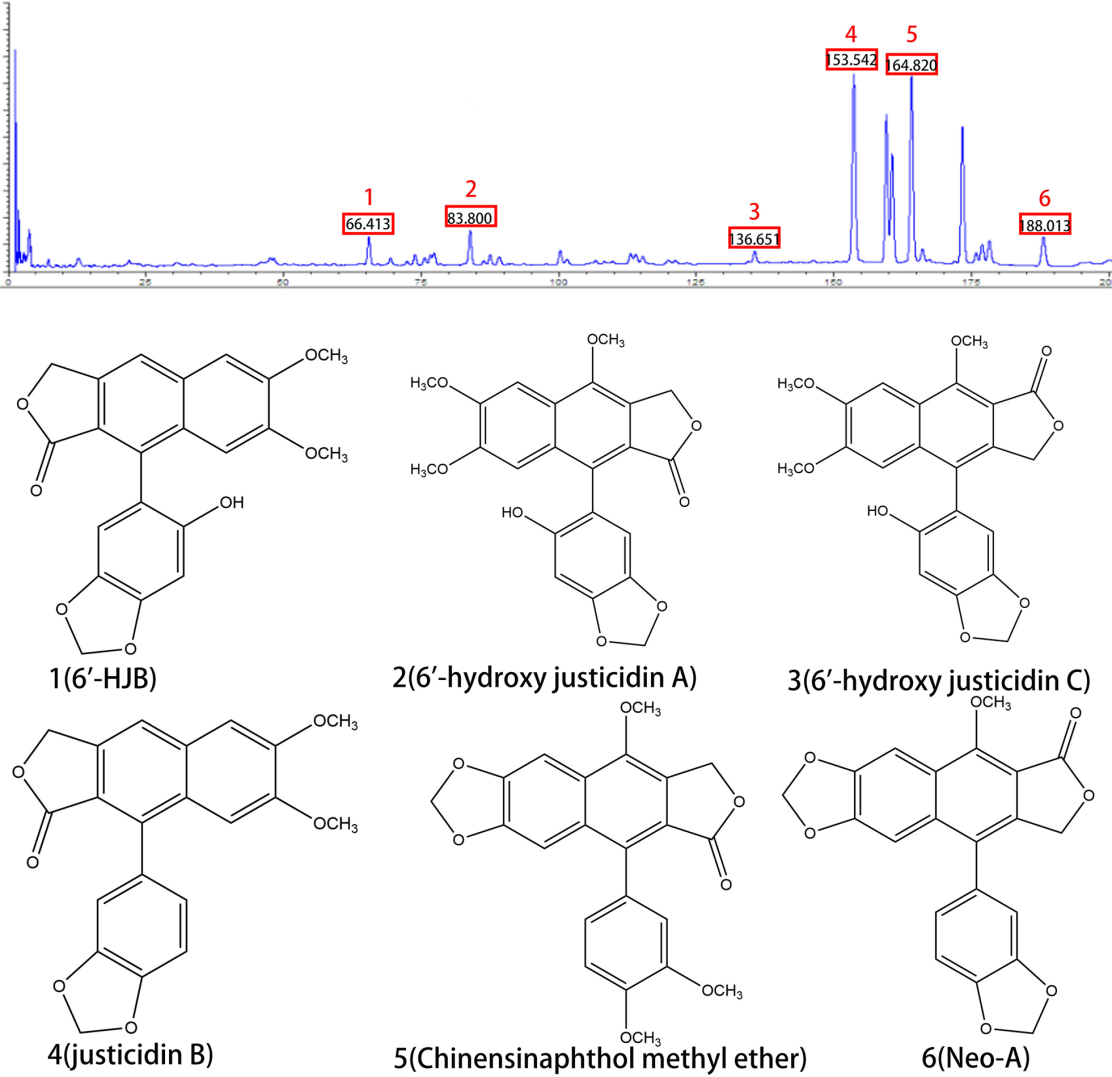
Analysis of three prototype components in serum samples by LC–MS. Liquid chromatogram: The chromatographic peak of the standard of the sample. Peak 1: 6'-HJB; Peak 2: 6´-hydroxy justicidin A; Peak 3: 6´-hydroxy justicidin C; Peak 4: justicidin B; Peak 5: Chinensinaphthol methyl ether; Peak 6: Neo-A

Using an optimized HPLC method, three prototype components were identified in serum samples collected 1 h after ethyl acetate extract administration (Fig. 3). Chromatographic separation was achieved on a Phenomenex Synergi™ C18 column (4 μm, 250 × 4.6 mm) with gradient elution of water (0.1% formic acid) and acetonitrile (0.1% formic acid) at 1.0 mL/min. The retention times (mean ± SD) were:

6'-HJB: 66.413 ± 0.3 min, Neo-A: 188.013 ± 0.5 min, Chinensinaphthol methyl ether: 164.820 ± 0.7 min. Peak identities were confirmed by comparing retention times and UV spectra with authentic standards. The method showed excellent linearity (R2 ≥ 0.999) across 0.1–100 μg/mL for all analytes, with intra-day precision (RSD ≤ 5.2%) and inter-day precision (RSD ≤ 7.8%).

LC–MS/MS analysis with multiple reaction monitoring (MRM) revealed measurable plasma concentrations of 6'-HJB and Neo-A at the peak time point (1 h post-administration): 6'-HJB: 2.1 ± 0.3 μM (n = 6), Neo-A: 1.8 ± 0.2 μM (n = 6).

Method validation parameters included: Recovery: 85.3–92.6%, Matrix effect: ≤ 12%, Lower limit of quantification: 0.1 μM.

These concentrations exceeded the in vitro IC₅₀ values of both compounds (2.5–18.3 μM), indicating their systemic exposure was sufficient to exert pharmacological effects.

### Activity evaluation in vitro

**Fig. 4:**
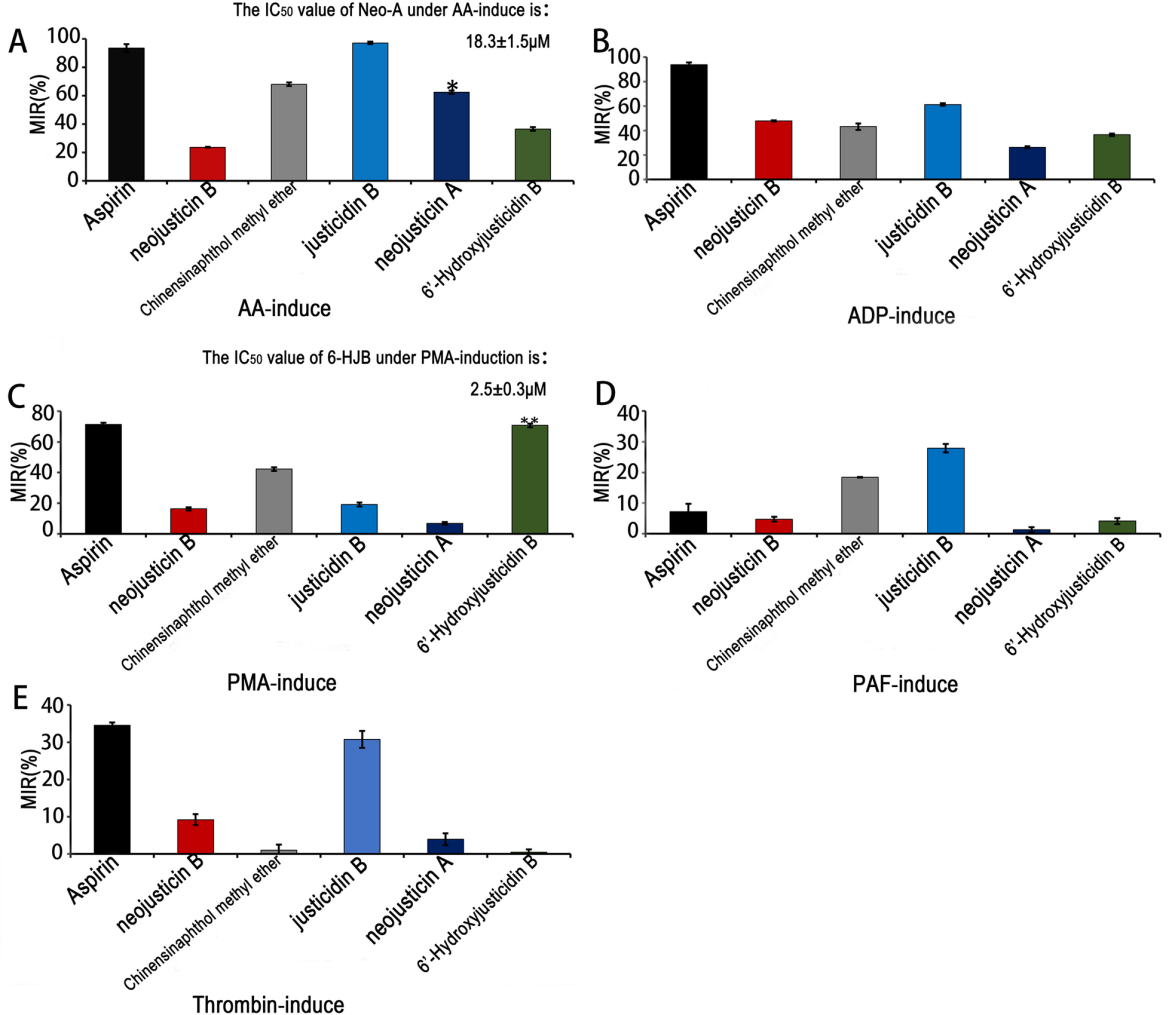
6'-HJB and Neo-A demonstrated stronger inhibition of PMA-induced platelet aggregation and AA-induced aggregation in vitro experiments, respectively. **A**–**E** The anti-aggregation of different compounds, *P < 0.05, **P < 0.01, ***P < 0.001

The antiplatelet activities of 6'-HJB and Neo-A were evaluated against multiple platelet agonists using the optical turbidimetric method (Fig. 4). Both compounds exhibited concentration-dependent inhibition, with 6'-HJB displaying preferential activity against PMA-induced platelet aggregation (IC₅₀ = 2.5 ± 0.3 μM, n = 3; inhibition rate = 70.69% ± 1.25% at 10 μM, P < 0.01 vs. control) (Fig. 4C). In contrast, Neo-A demonstrated stronger inhibition of AA-induced aggregation (IC₅₀ = 18.3 ± 1.5 μM, n = 3; inhibition rate = 62.40% ± 1.16% at 20 μM, P < 0.05 vs. control) (Fig. 4A).

Among the other tested 1-phenylnaphthalide lignans, justicidin B exhibited the most potent broad-spectrum antiplatelet effects, showing the strongest inhibition against AA-, ADP-, thrombin-, and PAF-induced aggregation, surpassing both Chinensinaphthol methyl ether and 6'-HJB in potency. Meanwhile, 6'-HJB displayed unique selectivity for the PMA pathway, suggesting a distinct mechanism of action.

For the 4-phenylnaphthalide derivatives, Neo-A (featuring a C-6/C-7 fused ring) exhibited significantly stronger AA pathway inhibition compared to neo-justicidin B (IC₅₀ = 18.3 ± 1.5 μM vs. 32.4 ± 2.1 μM, P < 0.05), whereas neo-justicidin B (with a C-6/C-7 -OCH₃ substitution) showed broader but weaker activity against other agonists.

To further validate the mechanism of action, Western blot experiments were conducted. Protein samples were collected from platelet cells treated with 6'-HJB and Neo-A at concentrations corresponding to their IC₅₀ values for PMA-induced and AA-induced aggregation, respectively. For 6'-HJB, the expression and phosphorylation levels of PKCδ were detected. The results showed that 6'-HJB treatment led to a significant decrease in the phosphorylation level of PKCδ, indicating its direct regulatory effect on PKCδ in the PMA pathway. Similarly, for Neo-A, the expression and activity-related markers of COX-1 were analyzed. Western blot results demonstrated that Neo-A treatment could modulate the expression and activity-associated modifications of COX-1, suggesting a direct regulatory role of Neo-A on COX-1 in the AA pathway.

These findings highlight the structural determinants of antiplatelet activity, with 6'-HJB and Neo-A emerging as promising candidates for targeted inhibition of specific platelet activation pathways.

### Proteomics and target identification

**Fig. 5:**
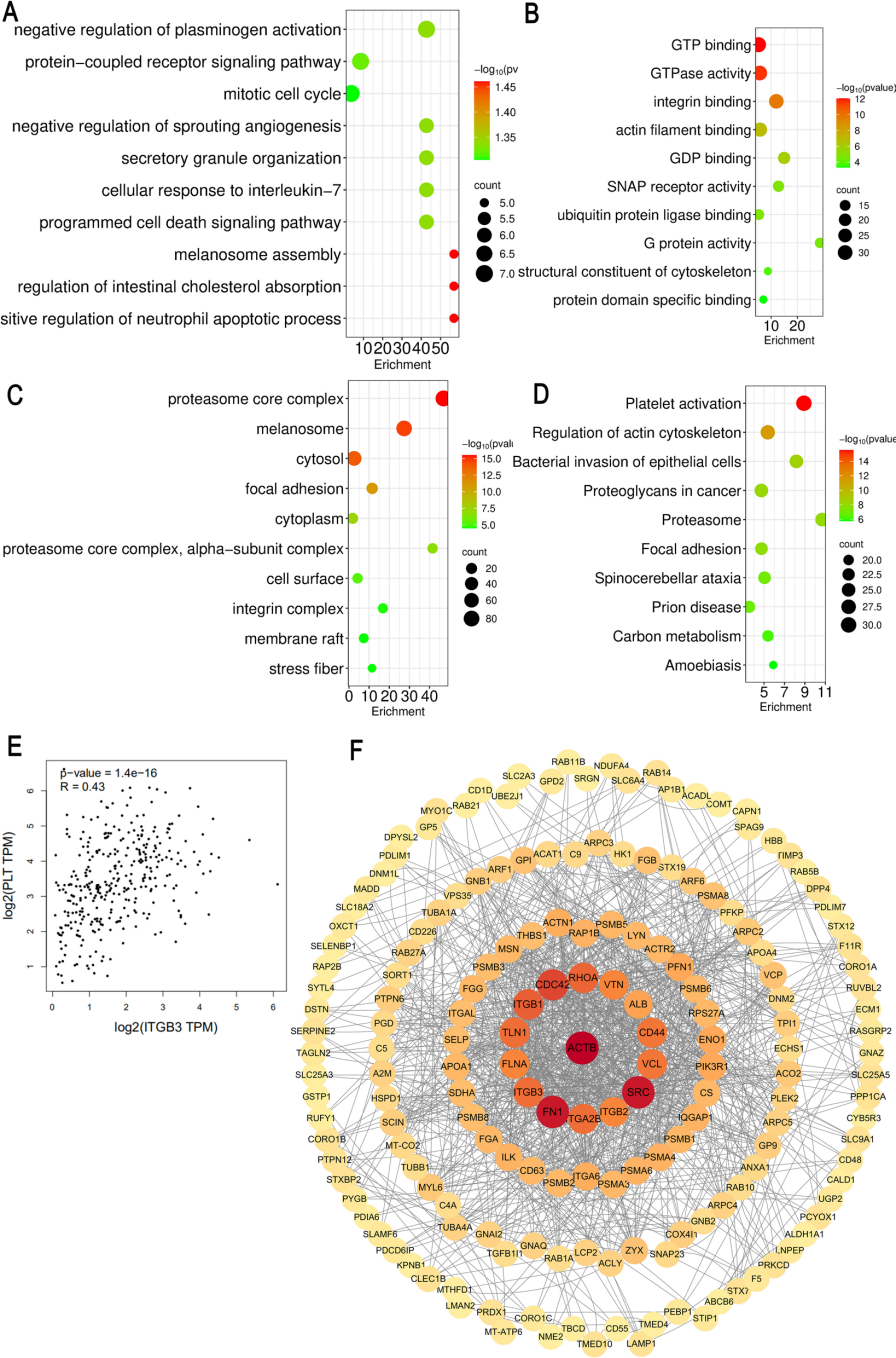
ITGB3 is a key target for antiplatelet effects. **A** Bubble chart of BP. **B** Bubble chart of MF. **C** Bubble chart of CC. **D** Bubble chart of KEGG. **E** ITGB3 expression positively correlated with platelet aggregation markers (r = 0.78, P < 0.001). **F** PPI network was constructed from proteins identified by the STRING database analysis

Proteomic analysis identified ITGB3 as a key differentially expressed protein in platelets treated with the ethyl acetate fraction. Using 2D-DIGE and MALDI-TOF–MS, ITGB3 was found to be upregulated by 2.3-fold (P < 0.001, n = 3 biological replicates) compared to control samples. Pathway enrichment analysis via DAVID revealed significant association with “Platelet activation” (hsa04611, P = 7.2 × 10⁻^4^) and “Thrombin signaling” (hsa04610, P = 1.5 × 10⁻^3^) (Fig. 5A–D), involving 12 interacting proteins (e.g., FAK, SRC) identified through STRING database analysis (Fig. 5F). GEPIA2 validation in GTEX-PLT dataset showed ITGB3 expression positively correlated with platelet aggregation markers (r = 0.78, P < 0.001), (Fig. 5E). Functional annotation indicated ITGB3 mediates platelet adhesion via interaction with fibrinogen, aligning with the observed antiplatelet effects of the ethyl acetate fraction. These findings collectively support ITGB3 as a critical target for the observed pharmacological activity.

### Molecular docking and MST validation

Molecular docking studies using AutoDock Vina showed that both 6'-HJB and Neo-A bind strongly to the ITGB3 (PDB ID: 9C1T). The docked poses exhibited different binding modes.

**Fig. 6:**
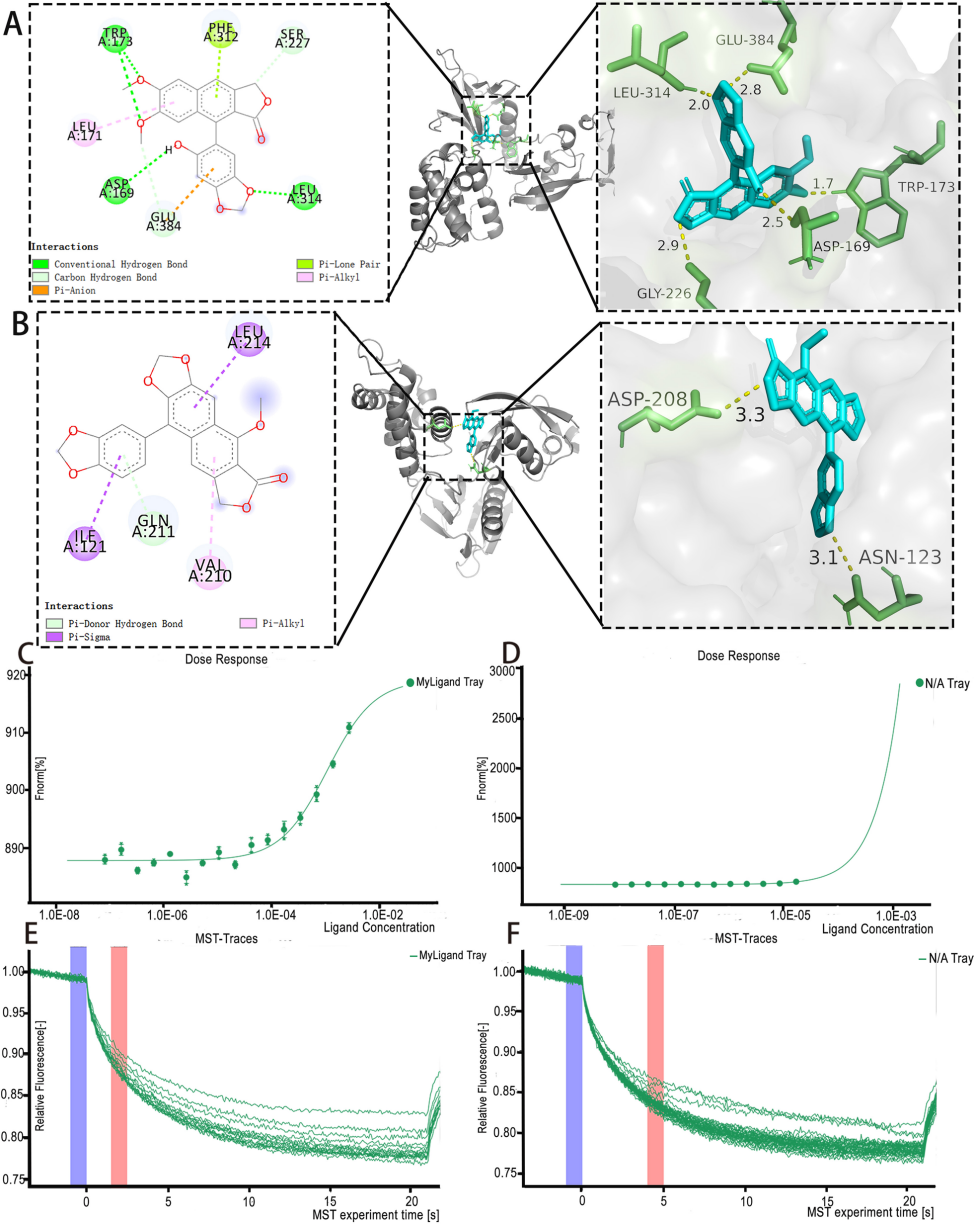
6'-HJB and Neo-A interacted with the α_IIb_β_3_ subunit of ITGB3 with high affinity.** A** 6'-HJB formed a hydrogen bond with TRP173/GLU314 of ITGB3 (ΔG = -7.11 kcal/mol. **B** Neo-A formed a hydrogen bond with ASP208 (ΔG = -5.9 kcal/mol). **C** ITGB3 (200 nM), 6'-HJB ranged from 2.62 mM to 8E-05 Mm, Kd = 0.0642 ± 0.005 μM. **D** Neo-A ranged from 16.7 μM to 0.00813 μM, Kd = 0.0097 ± 0.001 μM. **E–F** Molecular interactions of 6'-HJB and Neo-A by NT. 115 analysis

6'-HJB formed a hydrogen bond with TRP173/GLU314 and hydrophobic interactions with PHE312/LEU171, contributing to a binding energy of -7.11 kcal/mol (Fig. 6A). Neo-A formed a hydrogen bond with ASP208 and hydrophobic interactions with VAL210, resulting in a binding energy of -5.9 kcal/mol (Fig. 6B).

These results were validated by MST experiments, which measured direct binding affinities under physiological conditions (Fig. 6C–F). The Kd values were: 6'-HJB: 0.0642 ± 0.005 μM (n = 3), Neo-A: 0.0097 ± 0.001 μM (n = 3), Aspirin: 8.2 ± 0.7 μM (n = 3).

The observed Kd values were 128- and 845-fold lower than aspirin (Table 2), respectively, confirming the superior binding capacity of the isolated compounds. Binding curves were fit to a 1:1 Langmuir model (R2 ≥ 0.99), and nonspecific binding was < 5% as determined by buffer controls. These findings provide biophysical evidence supporting ITGB3 as the direct molecular target of the identified lignans.

**Table 2:** Kd and IC_50_ values of 6'-HJB, Neo-A vs. Aspirin

Compound	IC_50_ (µM)	Kd (µM)
6'-HJB	2.5 ± 0.3	0.0642 ± 0.005
Neo-A	18.3 ± 1.5	0.0097 ± 0.001
Aspirin	1–10	8.2 ± 0.3

## Discussion

In this study, the antiplatelet effects and molecular mechanisms of 6'-HJB and Neo-A from *Justicia procumbens* L. were systematically revealed for the first time [13]. The two lignans showed significant pathway selectivity: 6'-HJB specifically inhibited PMA-induced platelet aggregation via the 1-phenylphenacyl lactone structure and the hydroxyl group at the C-6 position (IC₅₀ = 2.5 μM), whereas Neo-A, with its 4-phenylphenacyl lactone skeleton and C-6/C-7 five-membered ring structure, was found to have the following antiplatelet effects [26]. 6/C-7 five-membered ring structure made it more favorable for the AA pathway (IC₅₀ = 18.3 μM) [27]. This finding is consistent with the conformational relationship of lignans reported in the literature: polar groups (e.g., hydroxyl groups) at the C-6 position enhance hydrogen bonding with PKCδ [28], whereas the five-membered ring structure may inhibit COX-1 activity through spatial site-barrier effects [29]. Notably, the PMA pathway inhibitory activity of 6'-HJB (IC₅₀ = 2.5 μM) was superior to that of the positive control quercetin (IC₅₀ = 12.3 μM), showing stronger target selectivity [30].

Proteomics and network pharmacology analyses consistently pointed to ITGB3 as the core target. Molecular docking reveals distinct binding mechanisms for 6'-HJB and Neo-A. The C-6 hydroxyl group of 6'-HJB anchors the ligand to ITGB3 through dual hydrogen bonds: a conventional bond with TRP173.HE1 (2.5–2.9 Å) and a carbon-hydrogen bond with GLU314.HN (1.7–2.8 Å), synergizing with hydrophobic interactions (PHE312/LEU171) to achieve high-affinity binding (ΔG = − 7.11 kcal/mol). In contrast, Neo-A lacks polar groups at C-6 but utilizes a C-6/C-7 fused furan ring to engage the VAL210-dominated hydrophobic pocket via Pi-Sigma interactions (3.1–3.3 Å), achieving moderate binding (ΔG = − 5.9 kcal/mol). This hydrophobic-driven mode resembles the clinical drug tirofiban (ΔG = − 8.8 kcal/mol) but lacks its ionic contributions, highlighting Neo-A’s potential for optimization. Future designs could hybridize these features—e.g., adding a hydroxyl to Neo-A’s furan—to bridge natural lignans and synthetic inhibitors, optimizing both specificity and efficacy. MST experiments further confirmed that the two have nanomolar affinity (Kd = 0.0097–0.0642 μM), which is 845-fold and 128-fold higher than that of aspirin, respectively, suggesting a stronger interaction with the target. Notably, ITGB3 expression was positively correlated with platelet aggregation function [31] (r = 0.78, P < 0.001), suggesting that inhibition of ITGB3 may be a new strategy for antithrombotic therapy [32].

This study establishes 6'-HJB and Neo-A from *Justicia procumbens* L. as the first-reported, highly potent natural inhibitors targeting integrin ITGB3—a pivotal receptor in platelet activation. While prior studies noted antiplatelet activity in the plant’s ethyl acetate extract, we now unequivocally isolate these lignans and demonstrate their role as novel, direct-acting ITGB3 antagonists with unprecedented inhibitory potency. Critically, MST analysis confirmed submicromolar binding affinity (Kd = 0.0642 μM for 6'-HJB; Kd = 0.0097 μM for Neo-A), with Neo-A achieving exceptional sub-10 nM affinity—a breakthrough for natural ITGB3 inhibitors.

This mechanistic discovery was enabled by our pioneering “proteomics-network pharmacology-MST” framework, representing a paradigm shift from phenotype-based natural product screening. The label-free MST technology further eliminated artifacts from protein immobilization (e.g., spatial hindrance in SPR), providing physiologically relevant target validation.

Both compounds exhibit outstanding ITGB3-specific antiplatelet effects, but their distinct pharmacokinetic profiles define complementary therapeutic advantages: 6'-HJB has high Caco-2 permeability (89.2%), positioning it as a prime candidate for oral formulation. Its remarkable safety profile (therapeutic index 84.3) surpasses montmorillonite (3.2) by 26-fold—setting a new benchmark for lignan-based inhibitors. Neo-A’s ultra-high affinity, strong plasma protein binding (92.7%), and CYP3A4-dependent metabolism support potential for sustained action via optimized dosing.

As the inaugural natural products identified as high-efficiency ITGB3 inhibitors, 6'-HJB and Neo-A offer groundbreaking leads for antithrombotic drug development. Our integrated methodology bridges target discovery, validation, and pharmacokinetic assessment—accelerating the translation of phytochemicals into clinically viable therapeutics.

## Conclusion

This study systematically elucidates the antiplatelet mechanisms of 6'-HJB and Neo-A from *Justicia procumbens* by integrating multi-omics, biophysical validation, and structure–activity analysis. We identified ITGB3 as the direct molecular target, with both compounds exhibiting nanomolar binding affinity (Kd = 0.0642/0.0097 μM) and pathway-specific inhibition of platelet aggregation (PMA- and AA-induced pathways, respectively). Their superior target selectivity, favorable pharmacokinetic profiles, and reduced bleeding risks compared to conventional therapies highlight their potential as novel ITGB3-mediated antithrombotic agents. This work establishes a target-driven framework bridging natural product research and drug development, advancing the translation of phytochemicals into precision therapies for thrombotic disorders. Future studies should focus on structural optimization and preclinical validation to further enhance efficacy and safety.

## Materials and methods

### Experimental animals

All experimental procedures were reviewed, approved, and supervised by the ethical board at South-central Minzu University (License No. SYSU-IACUC-2021-B1376). All procedures were performed according to the guidelines in the Guide for the Care and Use of Laboratory Animals.

Kunming breed mice, clean grade, body weight 20.0 ± 0.2–24 ± 0.2 g, male and female were supplied by Liaoning Provincial Laboratory Animal Resource Center (License No. SCXK (Liao) 2020–0001).

All experimental procedures were approved by the Animal Care and Use Committee of the Institute of Materia Medica, People’s Republic of China.

### Herbs and reagents

*Justicia procumbens* L. was authenticated by the Department of Traditional Chinese Medicine Identification of Hubei University of Traditional Chinese Medicine.

Adenosine diphosphate (ADP), arachidonic acid (AA), thrombin, platelet-activating factor (PAF), 12-O-tetradecanoylphorbol-13-acetate (PMA), collagen, epinephrine hydrochloride (Epinephrine hydrochloride), aspirin (Acetylsalicylic acid), and other reagents. Acetylsalicylic acid, dimethyl sulfoxide (DMSO), bovine serum albumin (BSA), and 0.5% carboxymethyl cellulose sodium (CMC-Na).

6'-HJB, Neo-A, justicidin B, Chinensinaphthol methyl ether, etc., were provided by the Provincial Key Laboratory of Traditional Chinese Medicine Resource and Traditional Chinese Medicine Chemistry, Hubei University of Traditional Chinese Medicine (purity ≥ 98%).

### Extraction and separation

*Justicia procumbens* L. herb (5 kg) was crushed, added with 80% ethanol (material-liquid ratio 1:10, v/w), and extracted under reflux at 80℃ for 3 times, each time for 2 h. The extracts were combined and concentrated under reduced pressure until it was free of alcohol flavor (temperature ≤ 50 °C, vacuum ≤ 0.08 MPa) to obtain the extract (yield: 12.3%).

The extract was suspended in distilled water (1:5, w/v), and liquid–liquid extraction was carried out sequentially with petroleum ether (3 × 2 L), ethyl acetate (3 × 2 L), n-butanol (3 × 2L), and water (3 × 2 L). Each extraction site was concentrated to dryness under reduced pressure, weighed, and stored under refrigeration.

### Platelet aggregation assay In vitro

After SD rats (n = 6) were anesthetized by intraperitoneal injection of 2% sodium pentobarbital (40 mg/kg), blood was collected from the abdominal aorta into a blood collection tube containing 3.8% sodium citrate (v/v 1:9). Whole blood was centrifuged at 200 × g for 10 min at room temperature (centrifuge model: DM0412, Shanghai Jinggong), and the upper layer of liquid was PRP; the remaining blood was centrifuged at 800 × g for 10 min, and the upper layer of liquid was PPP.

The platelet aggregometer (LBY-NJ4) was warmed up for 30 min, and the assay cups containing 300 μL of PPP and 260 μL of PRP samples were placed in the thermostat wells and warmed up at 37 °C for 1 min. Each channel was zeroed with PPP. 5% DMSO solution was used as a blank control. The reaction system was 260 μL of PRP + 30 μL of test sample (5% DMSO dissolved), incubated at 37 °C for 20 min, and then 10 μL of ADP (final concentration 10 μM) was added. The maximum aggregation rate (Aggregation %) was recorded within 5 min, and each group was measured three times in parallel, and the inhibition rate was calculated by the formula:

$${\mathrm{MIR}}\left( \% \right)\,\, = \,\,\left( {{1 - }\frac{{\text{MAR in treatment group}}}{{\text{MAR in control group}}} \, } \right) \times {1}00\% \,$$

### Coagulation time assay In vivo

During the experiment, healthy mice were randomly divided into six groups based on body weight: treatment groups J1, J2, J3, J4, J5, and a control group, with 10 mice per group (equal numbers of males and females). Mice were fasted for 12 h without water restriction before gavage. Treatment and control groups received 40 mL/kg via gavage, while the control group received 0.5% CMC-Na. Weight measurements were taken before the experiment. The dosage for each group was calculated based on the body weight of each mouse and administered orally in a single bolus ( The doses are as follows: 1.549 g/kg, 1.936 g/kg, 2.420 g/kg, 3.025 g/kg, 3.782 g/kg). After gavage, mice were allowed free access to food and water.

Kunming mice (n = 80) were randomly divided into 5 groups: blank control group (0.5% CMC-Na, 30 mL/kg), ethyl acetate extract low-dose group (11.8 mg/kg), medium-dose group (23.6 mg/kg), high-dose group (47.1 mg/kg), and positive control group (aspirin, 13.3 mg/kg).

Continuous gavage for 3 days, once a day, and fasting for 12 h after the last dose.1 h after the last dose, blood was collected from the retro-orbital venous plexus by placing a drop of blood on a slide (5 mm in diameter) and picking the drop of blood with a needle at 30 s intervals, and the time from blood collection to picking up the blood was recorded (coagulation time, s). Ten mice were tested in each group, and the results were expressed as mean ± standard deviation.

Based on the results from each dose, the LD_50_ value and 95% confidence limits were calculated using the modified Korth method. The calculated LD_50_ value was 2.4194 g/kg, equivalent to 201.617 g crude drug/kg. The 95% confidence limits ranged from 2.1050 to 2.7801 g/kg. The calculation formula is as follows:

$${\mathrm{m}}\,\, = \,\,{\mathrm{Xm}} - {\mathrm{I}}({\mathrm{p}} - 0.{5})$$

$${\mathrm{Sm}}\,\, = \,\,{\mathrm{I}}\left[ {\left( {\sum {\mathrm{p}} - \sum {\mathrm{p}}^{2} } \right)/{\mathrm{n}} - {1}} \right]^{{{1 \mathord{\left/ {\vphantom {1 2}} \right. \kern-0pt} 2}}}$$

The 95% confidence interval for m = m ± 1.96Sm.

In the formula, m represents the logarithm of LD_50_, Xm denotes the logarithm of the highest dose, I is the difference between the logarithms of adjacent dose groups, P indicates the mortality rate of each group, Ʃp is the sum of mortality rates across all groups, Sm is the standard error, and n is the number of animals per group.

By calculating the ED_50_ value through the dose–response curve, key parameters from the curve were: A (100% effect) = 140.4 ± 9.07 s at 47.1 mg/kg, and B = 92.1 ± 6.81 s. The 50% maximum effect (102.0 ± 0.07 s) was derived accordingly. The calculation utilized the following relationship:

$${\mathrm{ED}}_{{{50}}} \,\, = \,\,{\text{D - }}\frac{{\left( {{\text{A - 50\% of the maximum effect value}}} \right)*{\mathrm{X}}}}{{\mathrm{Y}}}$$

Where the dose interval X = 23.5 mg/kg (from C = 23.6 mg/kg to D = 47.1 mg/kg), the effect interval Y = 48.3 ± 2.26 s (from B to A), and Y' = 38 ± 9.00 s (the difference between the A value and the 50% effect value).

### Serum sample preparation

SD rats (n = 12) were divided into a blank group and a drug-administered group, 6 rats each. The rats were fasted without food and water for 12 h before gavage. Both groups were administered twice a day at an interval of 12 h for 3 consecutive days. A suspension of ethyl acetate extract of *Justicia procumbens* L. at a concentration of 97.20 g/mL was given to the administration group, and 0.5% CMC-Na was given to the blank group.

After 1.5 h of anesthesia with 2% pentobarbital sodium, 5 ~ 8 mL of blood was taken from the femoral artery. The blood was centrifuged at 3500 rpm for 10 min. The serum from each rat in the group was combined, and the supernatant serum was taken as 1.0 mL each, totaling 2 portions. The serums were extracted with 5 mL of acetone, vortexed and mixed for 5 min, and then left to stand for 5 min. 5000 r/min, and centrifuged at 4 ℃ for 10 min, and the upper layer of ethyl acetate layer was centrifuged into a centrifuge tube, evaporated in a water bath at 37 ℃ under nitrogen flow, and the residue was dissolved with 200 µL of acetonitrile, ultrasonicated for 5 min, and centrifuged for 10 min at 12,000 rpm/min. 20 µL of the supernatant was taken and analyzed by HPLC. Blank serum was processed in the same way and analyzed.

### Optimization of HPLC conditions

Mobile phase composition: water (phase A, containing 0.1% formic acid)—acetonitrile (phase B, containing 0.1% formic acid). Their high volatility is favorable for mass spectrometry detection. The gradient elution program was as follows: 0–130 min: 85% → 65% A, 130–170 min: 65% → 55% A, 170–220 min: 55% A, 20 μL (autosampler temperature: 4 °C).

Quality control was ensured by injecting mixed standards (6'-HJB and Neo-A) every 10 samples, requiring retention time RSD ≤ 2% and peak area RSD ≤ 5%.

An electrospray ionization (ESI) source operated in positive ion mode was employed. Key parameters were set as follows: capillary voltage 4500 V, nebulizer pressure 0.8 bar, drying gas flow rate 8 L/min at 200 °C, and mass scan range m/z 50—1600.

Quantification was performed by the external standard method, and the linear range of the standard curve was 0.1–100 μg/mL (r2 ≥ 0.999). Method validation demonstrated satisfactory precision (intra-/inter-day RSD ≤ 10%) and extraction recovery (≥ 85%).

### Activity screening of single compounds

6'-HJB, Neo-A, 6´-hydroxy justicidin A, 6´-hydroxy justicidin C, justicidin B, Chinensinaphthol methyl ether were dissolved in DMSO and prepared into a 10 mM masterbatch, which was diluted to the test concentration (0.1–100 μM) with PBS containing 0.1% BSA before use. The positive control, aspirin (10 mM), was also dissolved in DMSO and diluted.

Threshold activation amounts of each inducer (e.g., AA: 10.73 mM, ADP: 37.8 μM, Thrombin: 0.83 UN/mL, PAF: 13.52 μM, PMA: 6.55 μM) were determined by pre-experimentation to ensure that platelet aggregation was induced to 60% ± 5%.

Take 260 μL of PRP in the assay cup, add 30 μL of test compound (containing 0.05% DMSO), incubate at 37 °C for 20 min, and then add 10 μL of inducer. Platelet aggregation meter (LBY-NJ4) was used to record the change of transmittance within 5 min, and the Aggregation % and maximum inhibition rate (MIR) were calculated.

Each group of experiments was repeated 3 times, and the results were expressed as mean ± standard deviation (n = 3). The IC₅₀ value was calculated using the Logistic regression model in GraphPad Prism 9.0.

### Structural relationship analysis

1—Benzonaphthalenolide type: 6'-HJB (hydroxy substitution at C-6 position), justicidin B (methoxy substitution atC-6/C-7 positions). 4—Phenacolide type: Neo-A (five-membered ring at C-6/C-7), neojusticin B (methoxy substitution at C-6/C-7).

Compare the differences in the inhibition rates of AA (thromboxane A2 pathway) and PMA (PKC pathway) by different structural compounds. One-way analysis of variance (ANOVA) combined with Tukey's post-hoc test was used to verify the statistical significance of the structural differences in the activity (P < 0.05).

Using AutoDock Vina software, the compounds were docked with ITGB3 (PDB ID: 9C1T) to analyze the differences in binding modes and binding energies and to explain the effect of structure on affinity.

### Protein glue strip preparation and identification

Fresh PRP (1 mL) was lysed with RIPA buffer (50 mM Tris–HCl, pH 7.4, 150 mM NaCl, 1% NP-40, 0.5% sodium deoxycholate, 0.1% SDS) containing protease inhibitors (1 mM PMSF, 10 μg/mL aprotinin) using ultrasonication (30 s × 3, 40% power, ice bath). After centrifugation (12,000 × g, 15 min, 4 °C), the supernatant was subjected to BCA protein quantification. Proteins (50 μg/well) were separated by 10% SDS-PAGE (80 V for 30 min, 120 V for 90 min), stained with Coomassie Brilliant Blue R-250, and destained. Differential protein bands (≥ twofold change, p < 0.05 vs. control) were excised for in-gel trypsin digestion (1:50 w/w, 37 °C, 16 h) following reduction with DTT and alkylation with iodoacetamide. The resulting peptides were desalted using ZipTip C18, mixed with α-cyano-4-hydroxycinnamic acid matrix, and analyzed by MALDI-TOF–MS (Bruker UltrafleXtreme). MS data were processed using FlexAnalysis and searched against the Oryctolagus cuniculus Uniprot database using Mascot with trypsin digestion parameters (one missed cleavage allowed, mass tolerance ± 50 ppm, significance threshold p < 0.05).

### Network pharmacology and single-gene bioconviction analysis

#### STRING database analysis

Using the list of identified differential proteins (p < 0.05) as input, select the restriction “HOMOSAPIENS” to construct the protein interaction network (PPI network), download the obtained PPI network diagram in TSV format, and then construct the network diagram of the screened targets through the target-enriched pathway using Cytoscape 3.9.1 software. Meanwhile, the main active components with high degree values were screened for molecular docking confirmation.

#### GO/KEGG enrichment analysis

Differential proteins were uploaded to the DAVID v6.8 database and selected for GO Biological Process (BP), Molecular Function (MF), Cellular Component (CC), and KEGG pathways for enrichment analysis, setting p < 0.05 (Benjamini–Hochberg correction).

#### GEPIA database validation

The ITGB3 gene was retrieved from the GEPIA2 platform (https://gepia2.cancer-pku.cn), and the “Platelet” tissue type was selected to analyze the correlation between its expression and platelet count (PLT), mean platelet volume (MPV), etc. (Spearman's coefficient), and downloaded relevant datasets (e.g., GTEX-PLT) for validation.

### Molecular docking and MST experiments

#### Molecular docking

Molecular docking simulation using AutoDock Vina 1.1.2.

Download the crystal structure of ITGB3 (PDB ID: 9C1T) from the PDB database and remove the ligand and water molecules. Polar hydrogen atoms were added using PyMOL 2.5.2, and the energy was minimized using the AMBER14 force field (maximum 1000 iterations, convergence threshold 0.01 kcal/mol/Å).

The 3D structures of 6'-HJB and Neo-A were constructed by ChemDraw 21.0, and conformational optimization was carried out using the MMFF94 force field (convergence threshold 0.001 kcal/mol·Å). After generating SDF format files, they were converted to PDBQT format using AutoDock Tools 1.5.6.

Define the grid box centered on the active pocket of α_IIb_β_3_ subunit of ITGB3 (x: -10.2, y: 22.5, z: 5.8) with dimensions of 20 × 20 × 20 Å. The grid box was used to define the active pocket of the α_IIb_β_3_ subunit of ITGB3. A semi-flexible docking mode was used, with the receptor fixed and the ligand allowed to rotate. Twenty docked conformations were generated for each compound, and the conformations with the lowest binding energy were screened for analysis.

Redocking tests were performed on the known inhibitor aspirin (PDB ID: 3OGP) with a root mean square deviation (RMSD) of < 2 Å. The binding energy (ΔG) in units of ΔG was determined to be the lowest. Binding energy (ΔG) is in kcal/mol; negative values indicate stable binding.

#### MST assay

The recombinant human ITGB3 protein was obtained from Abcam and diluted in assay buffer containing 20 mM Tris–HCl (pH 7.5), 150 mM NaCl, and 0.05% Tween-20. Test compounds were prepared in a two-fold serial dilution ranging from 0.1 nM to 10 μM, with the final DMSO concentration maintained below 0.5% in all reactions. The protein-compound mixtures were incubated for 30 min at 25 °C under light-protected conditions prior to further analysis.

A Nano Temper NT.115 Pico was used with 50% LED power and 40% IR laser power. Each concentration point was tested 3 times, and the average value was taken. Curve fitting was performed using Nano Temper Analysis software, and Kd values were calculated using a 1:1 binding model. Negative control (buffer only) and positive control (known inhibitor 3-PO, Kd = 10 μM) were set.

## Data Availability

Data will be made available on request.
